# Primary Clear Cell Adenocarcinoma of a Urethral Diverticulum Treated with Multidisciplinary Robotic Anterior Pelvic Exenteration

**DOI:** 10.1155/2013/387591

**Published:** 2013-12-23

**Authors:** Dane Scantling, Curtis Ross, Jamison Jaffe

**Affiliations:** ^1^The Philadelphia College of Osteopathic Medicine, 4170 City Avenue, Philadelphia, PA 19131, USA; ^2^Hahnemann University Hospital, 230 North Broad Street, Philadelphia, PA 19102, USA; ^3^Albert Einstein Medical Center, 5501 Old York Road, Philadelphia, PA 19141, USA

## Abstract

Primary urethral carcinoma is extremely rare and is marked by a variety of clinical symptoms. Primary carcinoma of a urethral diverticulum is still rarer and clear cell adenocarcinoma of the urethra is particularly uncommon (Swartz et al., 2006). Such infrequency has led to inadequate management guidance in the literature for a disease that is often late in presentation and carries substantial morbidity and mortality. This treatable but grave disease deserves definitive curative treatment. We present the first published instance in which it was treated with robotic anterior exenteration. In our case, a 47-year-old female was referred to the urology service for investigation of recurring urinary tract infections. During the workup, the patient was found to have an advanced clear cell urethral adenocarcinoma originating in a urethral diverticulum. We discuss the natural history of this condition, its consequences, and the first instance of its treatment using robotic anterior pelvic exenteration.

## 1. Introduction

Carcinoma of the urethral diverticulum is an aggressive and uncommon affliction with a poor outcome. Clear cell adenocarcinoma is an especially rare cause of urethral carcinoma but is seen much more often within the setting of urethral diverticula than of other urethral cancers [1]. It presents with a variety of complaints but is often advanced at the time of diagnosis and is a difficult disease to treat as no clear consensus exists. With the increasing use of robotic surgery, more options are now available than when the disease was first described and literary evidence now bears the burden of evaluating such new modalities. In our knowledge, we report the first known case of a primary clear cell adenocarcinoma of a urethral diverticulum treated with robotic anterior exenteration and discuss the important features of this rare disease and treatment.

## 2. Case Report

**Figure 1:**
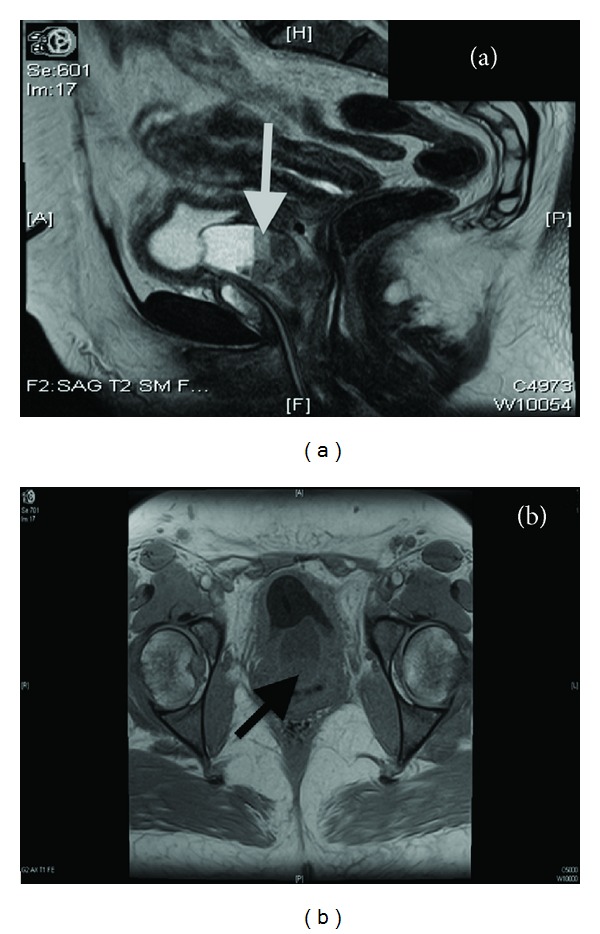
Magnetic resonance imaging shows 4.4 cm × 3.2 cm × 3.6 cm nodular enhancing mass concerning malignancy.

A 47-year-old African American female with a chronic history of recurrent urinary tract infections was referred to our urology service after a diagnosis of urethral carcinoma was made by her primary physician during the investigation of gross hematuria, hesitancy, straining, and urge incontinence. Additional medical conditions included only hypertension. Cystoscopy was significant for a sizeable papillary urethral mass emanating from a urethral diverticulum, the biopsy of which yielded a diagnosis of clear cell adenocarcinoma. A contrast enhanced MRI of the pelvis revealed a 5.1 cm × 4.3 cm × 4.0 cm urethral diverticulum containing a 4.4 cm × 3.2 cm × 3.6 cm nodular enhancing mass concerning malignancy (Figures 1(a) and 1(b)). The MRI also showed enlarged pelvic sidewall lymph nodes of up to 1.2 cm on the right and 1 cm on the left (Figures 1(a) and 1(b)). A CT scan of the abdomen and pelvis without and with contrast was obtained; it revealed a 4 cm × 4 cm × 3.5 cm ill-defined urethral mass and enlarged pelvic lymph nodes of up to 2 cm. A CT scan with contrast of the chest and bone scan both revealed no metastasis.

The case was discussed with the patient as well as with a multidisciplinary tumor board, ultimately resulting in the decision to undertake a robotic assisted radical anterior exenteration with Indiana pouch creation involving the urology, gynecologic oncology, and general surgery teams.

Preoperative vital signs were a blood pressure of 124/74 mmHg, pulse of 72/minute, respiratory rate of 18/minute, and a BMI of 31.8. Urinalysis revealed only trace blood, a specific gravity of 1.015, and a pH of 5.5. Flagyl 500 mg PO and neomycin sulfate 500 mg PO were initiated preoperatively.

**Figure 2:**
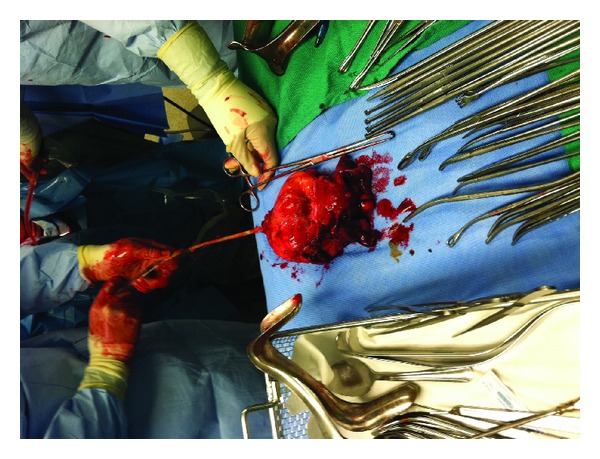
The surgical specimen removed transvaginally included the bladder, uterus, cervix, bilateral ovaries, fallopian tubes, and urethra as well as the anterior vagina.

The urological service initiated the operation using a DaVinci robot to accomplish a radical cystectomy, hysterectomy, and urethrectomy. After robotic dissection of the bladder to the urethra, the posterior aspect of the vagina was entered using the monopolar shears in a cut fashion. This was then taken down on either angle laterally. Once this was completed, attention was then turned to the vagina from a vaginal approach by the gynecology service. The posterior part of the vagina was identified and grasped. It was then taken out to the pelvic sidewall and, using a Bovie electrocautery device, the vagina was then transected laterally to the vaginal sidewall on either angle giving a wide margin around the suburethral mass. Once this was complete, using the Bovie, the vagina was taken off anteriorly at the level of the pelvic outlet. This was then transected including the urethra, just inside of the vagina. The freed bladder, uterus, cervix, bilateral ovaries, fallopian tubes, and urethra as well as the anterior vagina were removed transvaginally by the gynecology service as shown in Figure 2.

Once completed, pneumoperitoneum was reaccomplished using a latex glove and sponge placed into the vagina. A full extensive pelvic lymph node dissection was then done bilaterally. At this stage, robotic assistance was terminated and the gynecology team reconstructed the vagina. The posterior vagina was then connected in an uninterrupted fashion with four sutures to the anterior aspect of the vagina. The lateral aspects of the vagina were reattached in a running locking fashion to create a vagina with a depth of 3-4 cm without leakage.

The general surgery service then commenced their portion of the case with 6 cm subumbilical incision and placement of a hand port and laparoscopic trocars placed where the robotic trocars had been. The colon was mobilized using hand-assisted laparoscopic technique and the pneumoperitoneum was then desufflated; the hand port was removed and the ascending colon, including the terminal ileum and transverse colon, was exteriorized. The terminal ileum was divided 20 cm from the ileocecal valve using an Endo-GIA stapler. Transverse colon was divided similarly just distal to the hepatic flexure. The transected terminal ileum and transverse colon were brought into apposition and an ileocolic anastomosis was created using an Endo-GIA stapler.

At this point, the left ureter, which had been clamped during the robotic portion of the case, was mobilized and dissected up to the level of the renal pelvis. Once isolated, it was mobilized behind the renal sigmoid colon. The right colon was minimally mobilized due to the patient's decision to have her stoma on the right side. The right colonic specimen was subsequently utilized for creation of the Indiana pouch, which was created by suturing the antimesenteric border along the right colon to itself and incising using electrocautery. The catheterizable channel, approximately 10 cm of ileum, was then imbricated. A 12-French silicone catheter was passed through the 10 cm segment, through the ileocecal valve and into the right colon. The channel was then tapered using an endovascular stapler. The ileocecal valve was then imbricated to prevent kinking. The ureters were introduced through the dependent portion of the pouch and good urine efflux was noted after the clamps were removed. Both were secured and had 6-French J stents placed with good return of urine. The areas around each insertion site were imbricated to prevent reflux. An 18-French cecostomy tube was placed in the dependent portion of the Indiana pouch and brought through the skin. A leak test using 600 mL of water was negative and a 12-French catheter was used without difficulty. The neobladder was then secured in the posterior peritoneal sheath to prevent any twisting. The stoma was subsequently secured without difficulty.

The total case time was 8.5 hours. The total estimated blood loss for the entire procedure was 350 mL. The total crystalloid infusion was 7 L. No other fluids were required. Pathology of the specimens revealed the following: A 3 cm × 1.6 cm × 1.2 cm grade 2 clear cell urethral adenocarcinoma within a urethral diverticulum invading the anterior vagina with negative margins, distal bilateral ureters with negative margins, 21 negative lymph nodes, and a negative hysterectomy specimen. Pathologic staging was pT3N0M0.

The patient was placed in the ICU following the operation, extubated, and continued to exhibit stable vital signs. She was subsequently transferred out of the ICU, was able to ambulate slowly, and had her nasogastric tube removed. After several days on the general medical floor, her postoperative ileus resolved and her Indiana pouch continued to function well. However, on the second week of admission and while still being on the general medical floor, she again had symptoms of an ileus. A replaced nasogastric tube provided symptomatic relief and, after three days, was removed with initiation of a clear and then regular diet which she tolerated well. On the third week of admission, the patient was noted to have a deteriorating mental status and was transferred to the SICU. A CT scan showed evidence of a moderate amount of blood products contained within the abdomen consistent with a liver laceration felt to be most likely due to retractor use during the initial operation. Once stabilized with a transfusion of 2 units of packed red blood cells, she was transferred back to the general medical floor and continued to recover without additional complications. Her JP drain was removed and she began to irrigate her own Indiana pouch twice per day in addition to catheterizing the pouch two to three times per day. Home care therapy, rehabilitation, and nursing were arranged and the patient was cleared for discharge on postoperative day twenty. Adjuvant therapy was subsequently discussed but declined by the patient.

The patient was most recently followed up one year postoperatively. At that time, she reported that she remains sexually active and has her normal bowel habits. She also reports catherizing her pouch without difficulty.

## 3. Discussion

Primary urethral carcinoma is an extremely rare condition in the United States and primary carcinoma of a urethral diverticulum is still rarer [2]. Such infrequency contributes to a lack of treatment options and understanding of the disease. Age adjusted incidence for primary urethral cancer has been found to be 4.3 per one million men and 1.5 per one million women in the United States [2]. This incidence increases with age to a maximum of 32 cases per one million Americans how aged 75–84 years [2]. Incidence in both males and females is higher in African Americans than in Caucasians [2]. Regardless of this, the disease remains uncommon with primary urethral carcinoma representing only 0.02% of female malignancies [1, 3]. Of the total female population, about 1–6% have a urethral diverticulum and primary urethral diverticular carcinomas are even more uncommon with only about one hundred reported cases since 1951 [1, 4]. Tumors arising from urethral diverticulum represent only 0.002% of female malignancies [4]. While different studies have found substantial variance, the vast majority of primary urethral cancer in either sex and any race is squamous cell carcinoma as it represents almost twice as many cases as transitional and adenocarcinoma combined [2, 4, 5]. Clear cell adenocarcinoma, however, has a clear association with diverticula and is the most common malignancy arising from them [1, 4]. While only 10% of urethral cancer is clear cell adenocarcinoma, a third of such cancers originates within a diverticulum [1].

The urethra consists of five distinct tissue layers; the mucosa, submucosa, and three muscular layers [6]. Diverticulum in the urethra, like those in the gut, is outpouching of mucosa. Distally, this mucosa is nonkeratinized stratified squamous epithelium [6]. Proximally, it becomes transitional cell in nature as it nears the bladder neck [6]. In the urethra, the cause of diverticula is often unknown but encompasses acquired and congenital subtypes. It has been hypothesized that rupture of infected periurethral glands into the urethral lumen may be responsible for most acquired cases [6, 7]. This is supported by the prevalence of clear cell adenocarcinoma arising from diverticula and may be of particular interest here, as our case involved a female presenting initially with recurring urinary tract infections.

Differentiation of primary origin with urethral carcinoma subtypes can be challenging, such disease remains quite rare. In women, the short urethra of the female leads to rapid encroachment of extending bladder transitional cell carcinoma past the bladder neck. In the case of clear cell of the urethra, local extension may mimic a primary clear cell adenocarcinoma of the vagina and differentiation is important for proper treatment.

In about half of patients, urethral carcinoma presents first with signs of either obstructive or irritative voiding and presents, in about a quarter of patients, urethral bleeding [3]. This may lend the disease to first discovery with subsequent investigative cystourethroscopy. As signs and symptoms are often nonspecific and prognosis can be poor, proper diagnostic methodology is important. Intravenous pyelography, voiding cystourethrography, CT, MRI, and cystourethroscopy may all be pivotal in attaining a diagnosis [5]. In several studies, the neoplasm was noted as a soft tissue mass located caudally to the bladder on CT or MRI [5]. Cystourethroscopy with biopsy is key to a proper and definitive diagnosis [5].

Given the short urethra found in females, an already aggressive disease has substantial chance of local extension and major complications. Indeed, 55% of urethral carcinoma recurs despite treatment and 10-year survival remains at only 60% [3]. Clinically, palpable lymph nodes are found in about third of patients and more than 90% are metastatic at diagnosis [7]. Clear cell adenocarcinoma remains a grave diagnosis but has the most favorable prognosis of the three most common urethral cancers with 54% experiencing disease free survival with any treatment modality and a disease free survival rate of 73% when treated with anterior exenteration alone [5]. Other treatment options for diverticular carcinoma include diverticulectomy alone, radiation alone, or diverticulectomy with radiation [5]. These carry disease free survival of 33%, 20%, and 50%, respectively, with anterior exenteration alone yielding the best opportunity for a positive long-term outcome [5]. Accordingly, this is an aggressive but treatable disease requiring aggressive treatment which, in this case, was robotic anterior pelvic exenteration. Pelvic exenteration is a rigorous and invasive procedure with its own substantial morbidity and mortality. As such, it is viewed as a treatment reserved for last resort when no other recourse exists to obtain a cure [8]. It was first described in 1948 by Dr. Alexander Brunschwig at Memorial Hospital for use in recurring or persistent gynecological malignancy, primarily for cervical cancer but more rarely for vulvar, endometrial, and ovarian cancer of the central pelvis [8]. While the procedure itself has been slow to change, mortality from the operation itself has dropped from an initial 23% to 0–5.3% as surgical technique and patient selection have drastically improved [9].

Further improvement may be afforded by the use of robot assisted minimally invasive surgery [10, 11]. A methodology that has been increasingly used with success in a variety of pelvic applications, including prostate cancer and mesorectal excision, and similar oncologic results can be expected in anterior exenterations [12]. Numerous studies propose varying levels of benefit over open techniques regarding mortality, morbidity, blood loss, hospital stay, and an increased ability to visualize the deep pelvis without sacrificing oncologic results [11, 13–15]. This is of particular value in procedures already carrying substantial risk of complications and death such as total anterior exenteration, but studies of robotic exenterations typically have few patients and thus low study power.

Our case is the first reported of clear cell adenocarcinoma of a urethral diverticulum treated with robotic exenteration. As few primary diverticular carcinomas have been reported, treatment has been inconsistent. Only one case of diverticular cancer, an adenocarcinoma, is known to have been treated with an anterior exenteration [5]. This instance was done in an open fashion. Our outcome was ultimately favorable, as anticipated, but the patient did suffer several unforeseen complications. The substantial morbidity provided by the incidental liver laceration remains a reminder of the significant dangers of such invasive surgery, even when done robotically. Other incurred complications were relatively minor: a protracted hospital stay and postoperative ileus. In our particular case, we demonstrated that this operation is possible using robotic technology and a multidisciplinary approach, but we did not demonstrate the particular benefits typically associated with robotic surgery. With the advancement of robotic technique and accompanying surgeon technique, we see an increasing role for the use of robotic exenteration in the treatment of advanced pelvic malignancies, including primary urethral diverticular clear cell adenocarcinoma. As in any case report, further study is needed to evaluate this modality.
